# Pseudomonocytosis on a Sysmex XN haematology analyser masking the monocytopenia of hairy cell leukaemia in a South African woman

**DOI:** 10.4102/ajlm.v14i1.2617

**Published:** 2025-03-25

**Authors:** Stephanie J. Kennedy, Anne-Cecilia van Marle

**Affiliations:** 1 Department of Haematology and Cell Biology, Faculty of Health Sciences, University of the Free State, Bloemfontein, South Africa; 2 Department of Haematology, National Health Laboratory Service, Bloemfontein, South Africa

**Keywords:** hairy cell leukaemia, pseudomonocytosis, monocytopenia, haematology full blood count analyser, Sysmex XN-series

## Abstract

**Introduction:**

Hairy cell leukaemia (HCL) is a rare B-cell lymphoproliferative disorder characterised by medium-sized villous lymphocytes (‘hairy cells’) and monocytopenia in the peripheral blood. Automated full blood count (FBC) haematology analysers may spuriously count ‘hairy cells’ as monocytes, resulting in pseudomonocytosis.

**Case presentation:**

A 72-year-old woman presented with symptomatic anaemia and massive splenomegaly to a regional hospital in North West province, South Africa, in June 2023. An FBC and differential count, performed on a Sysmex XN-series haematology analyser, revealed a monocytosis of 42.82 × 10^9^/L. However, a manual differential count, peripheral blood microscopy, and multiparameter flow cytometry confirmed a monocytopenia with numerous ‘hairy cells’.

**Management and outcome:**

The patient was referred to a tertiary hospital where bone marrow morphology and a *BRAFV600E* mutation confirmed a diagnosis of HCL. Unfortunately, she demised shortly after admission.

**Conclusion:**

Here, we report a case of HCL where a Sysmex XN-series artifactually counted ‘hairy cells’ as monocytes, masking the characteristic monocytopenia. With the recent introduction of Sysmex XN-series FBC haematology analysers (Sysmex Corporation, Kobe, Japan) in National Health Laboratory Service laboratories across South Africa, we urge operators to be cognisant of the inherent limitations of FBC analysers in generating blood counts.

**What this study adds:**

Even modern automated laboratory analysers with advanced technologies have inherent limitations. This case highlights the importance of a manual differential count and peripheral blood smear review in the era of automation.

## Introduction

Hairy cell leukaemia (HCL) is a rare B-cell lymphoproliferative disorder accounting for approximately 2% of lymphoid leukaemias, and usually involves the bone marrow (BM), splenic red pulp, and peripheral blood (PB).^1^ Although lymphocytosis is uncommon in HCL (5% – 10%), circulating ‘hairy cells’, albeit at low numbers, are characteristic (85%).^1^ Variable cytopenias are a frequent laboratory feature, whilst monocytopenia is almost always present (98%).^1,2^

An accurate automated white blood cell (WBC) differential count demonstrating monocytopenia should trigger a suspicion of HCL and warrants careful morphologic assessment for ‘hairy cells’.^3,4^ However, certain automated full blood count (FBC) haematology analysers may misidentify ‘hairy cells’ as monocytes, resulting in pseudomonocytosis.^5,6^

In 2022, the National Health Laboratory Service (NHLS) and Roche Diagnostics (Pty) Ltd entered into a Service Level Agreement for the placement of Sysmex XN-series automated FBC haematology analysers (Sysmex Corporation, Kobe, Japan) in NHLS laboratories (*n* = 146) across South Africa. This national roll-out involved the placement of the Sysmex XN-1000 series in medium (*n* = 51) and large laboratories (*n* = 20) and XN-L 530 instruments in smaller laboratories (*n* = 75).^7^

Automated FBC haematology analysers generate valuable quantitative and qualitative information that can assist laboratory staff with the accurate interpretation of FBC results, as well as alert them to prioritise abnormal samples for PB smear review.^8,9^ Not all NHLS laboratories are staffed with pathologists. Therefore, technologists and technicians frequently rely on automated analyser data to guide referral for microscopy.

## Ethical considerations

Ethical clearance was obtained from the Health Science Research Ethics Committee of the University of the Free State (reference no.: UFS-HSD2024/1295). The patient’s results were pseudonymised to ensure patient confidentiality and stored securely on a password-protected computer only accessible by the authors. Attempts to locate the patient or their next of kin to obtain permission to publish this report were unsuccessful.

## Case presentation

A 72-year-old woman presented with symptomatic anaemia to a regional hospital in North West province, South Africa, in June 2023. On clinical examination, she had massive splenomegaly but no lymphadenopathy. An FBC was performed on a Sysmex XN-1000 series and showed pancytopenia (haemoglobin 2.9 g/dL, platelet count 97 L × 10^9^/L, neutrophils 0.93 × 10^9^/L), with a leucocytosis of 58 × 10^9^/L. The automated WBC differential count showed a lymphocytosis of 14.16 × 10^9^/L and a marked monocytosis of 42.82 × 10^9^/L. The PB smear was referred to the NHLS Universitas Academic Laboratory, South Africa, for pathologist review.

**FIGURE 1:**
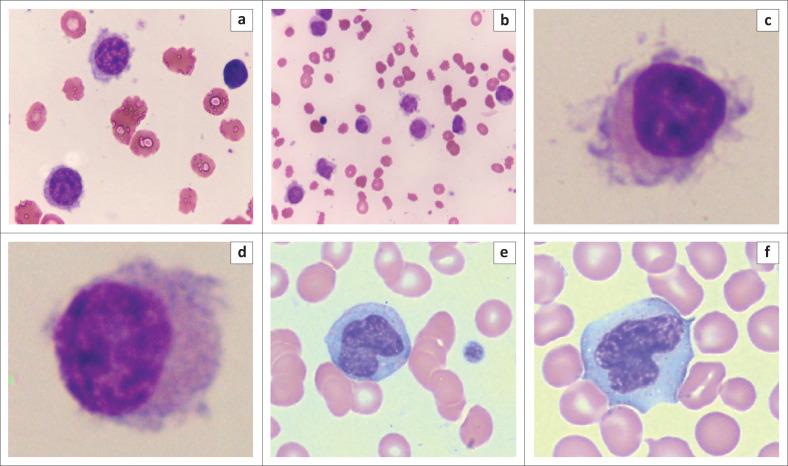
(a–d) ‘Hairy cells’ on the peripheral blood smear of a 72-year-old woman diagnosed with hairy cell leukaemia at National Health Laboratory Service Universitas Academic Laboratory, South Africa, in June 2023. Wright’s stain (a) 50× objective, (b) 10× objective and (c, d) 100× objective. (e, f) For comparison, images of normal monocytes on a peripheral smear are shown; Wright’s stain, 50× objective. These cells lack the circumferential villi and ground-glass chromatin pattern of ‘hairy cells’.

A manual differential count and PB smear microscopy revealed almost no monocytes. However, abnormal lymphocytes were increased (78%) (Figure 1). These cells were intermediate in size, with abundant pale blue cytoplasm and circumferential villi. Their nuclei were kidney-shaped with a ground-glass chromatin pattern and inconspicuous nucleoli.

**FIGURE 2:**
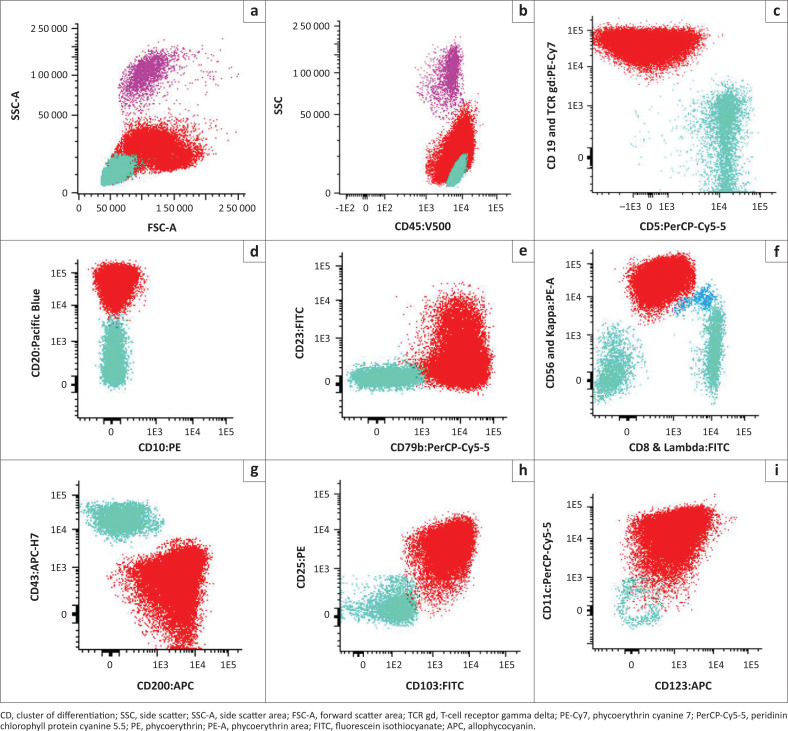
Flow cytometry dot plots of the pertinent cluster of differentiation markers used in the immunophenotypic diagnosis of hairy cell leukaemia that were performed for our patient at National Health Laboratory Service Universitas Academic Laboratory, South Africa, in June 2023. The population of interest is demonstrated in red. The teal green population represents normal T cells, whilst the pink population constitutes neutrophils, and the blue population constitutes natural killer cells. (a, b) The hairy cells are large in size (increased FSC-A) with bright CD45 expression. (c–f) The hairy cells brightly express pan-B-cell markers, CD19, CD20, and CD79b, with surface kappa light chain restriction, confirming clonality. (g–i) The hairy cells express CD200 with co-expression of villous markers, CD11c, CD25, CD103, and CD123.

Multiparameter flow cytometry was performed on the FACSCanto™ II (Becton, Dickinson, and Company, Franklin Lakes, New Jersey, United States), and the data analysed on Infinicyt™ version 2.0 (Cytognos, Santa Marta de Tormes, Salamanca, Spain) software. Analysis revealed an abnormal population of large cells with moderate complexity and bright CD45 expression extending into the ‘monocyte window’ (Figure 2). These cells expressed pan-B-cell markers, CD19, CD20, CD22, CD79b, and sIgM, with surface kappa light chain restriction. Villous markers (CD11c, CD25, CD103, CD123) and CD200 were positive. This immunophenotype is characteristic of HCL.^2,6^ There was no significant monocyte population.

Because monocytopenia, rather than monocytosis, is typically seen in HCL, an FBC and automated WBC differential was repeated on the Advia® 2120i (Siemens Healthineers, Erlangen, Germany) FBC analyser in our laboratory. The same blood sample subsequently revealed a monocytopenia of 0.93 × 10^9^/L, with an increase in the lymphocyte count to 34.51 × 10^9^/L. These counts correlated with the pathologist’s manual differential count and flow cytometry analysis. It was concluded that the Sysmex XN-1000 series spuriously counted the ‘hairy cells’ as monocytes, resulting in pseudomonocytosis.

## Management and outcomes

The patient was referred to a tertiary hospital. Further workup of the case confirmed a diagnosis of HCL, with the detection of the *BRAFV600E* mutation and characteristic BM morphology. Unfortunately, the patient demised shortly after admission, and further details regarding treatment could not be obtained.

## Discussion

Monocytopenia in the setting of pancytopenia should trigger a suspicion of HCL.^3,4^ Demonstrating clonal B cells with ‘villous’ markers (CD11c, CD25, CD103, and CD123) on multiparameter flow cytometry is critical for the timely diagnosis of HCL.^2,6,10^ However, characteristic lymphocytes with circumferential villi (‘hairy cells’) may be sparse on PB morphology.^1,10^ Unless the pathologist has a high index of suspicion on the initial PB smear review, the diagnosis of HCL may be unnecessarily delayed until a BM aspirate and trephine biopsy and molecular studies for the *BRAFV600E* mutation are performed. Furthermore, BM aspiration is frequently unsuccessful because of BM fibrosis in HCL.^2,10^ It is important to note that the leucocytosis and abundance of ‘hairy cells’ (78%) that were seen in our case is unusual. Lymphocytosis is only reported in 5% – 10% of cases of HCL.^1^ By comparison, the HCL cases evaluated by Tofan et al.^3^ and Furundarena et al.^11^ revealed an average of 19.6% and 9.4% circulating ‘hairy cells’.

Villous lymphocytes are not unique to HCL. Their differential diagnosis includes artefact and other splenic B-cell lymphomas/leukaemias, namely splenic diffuse red pulp lymphoma, splenic marginal zone lymphoma and HCL variant (an entity retained in the International Consensus Classification, but reclassified as splenic B-cell lymphoma/leukaemia with prominent nucleoli in the World Health Organization Classification of Haematolymphoid Tumours 5th edition).^12^ Considering its relative sensitivity and specificity for HCL, the presence or absence of monocytopenia is often relied on during the initial assessment of these lymphomas to assist with the differential diagnosis.^3,10^ However, the sensitivity for detecting monocytopenia in HCL varies amongst automated FBC haematology analysers, and may be more reliable on instruments using peroxidase staining techniques such as the Advia® 2120i.^4^ Instruments utilising fluorescent staining and light scatter, or physical properties of cells, may yield inaccurate monocyte counts in HCL.^3,4^

Sysmex XN-series automated FBC haematology analysers incorporate cytochemical reactions followed by fluorescence flow cytometry using a semiconductor laser to perform a WBC differential count. This method involves the addition of a fluorescent dye to WBCs, which are subsequently separated by their different light scatter properties according to size (forward scatter), complexity (side scatter), and fluorescence signal.^3,13,14^ Scattergrams provide a visual representation of separated cell population in the white cell nucleated-, white cell differential-, and white cell precursor channels.^3,13^ Additionally, internal protocol messages or ‘flags’ assist with the rapid identification of quantitative and qualitative WBC abnormalities.^8,9^

**FIGURE 3:**
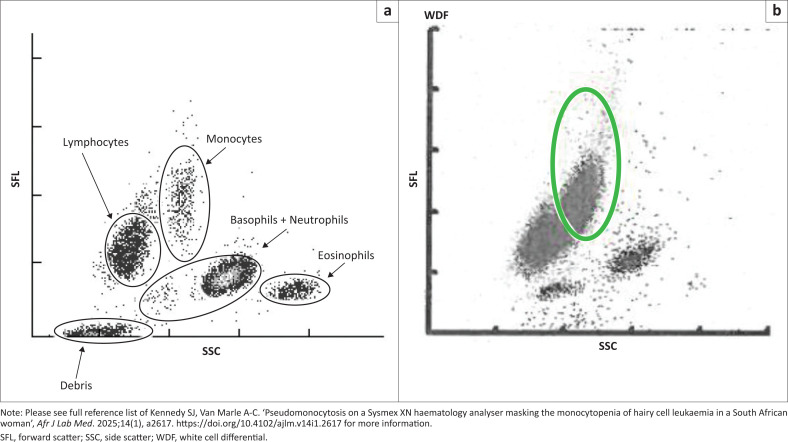
(a) The white cell differential channel on the XN-series (Sysmex Corporation, Kobe, Japan) primarily classifies white blood cells. This normal scattergram displays lymphocytes, monocytes, eosinophils, basophils, neutrophils, and debris.^13,14^ (b) The white cell differential scattergram for our case, performed on a Sysmex XN-1000 at a regional laboratory in North West province, South Africa in June 2023, demonstrated a large population of cells in the ‘monocyte window’.

In our case, the Sysmex XN-1000 series correctly flagged ‘neutropenia’, ‘leucocytosis’, ‘lymphocytosis’ and the presence of ‘blasts/abn lymphocytes?’ (sic). The white cell differential scattergram showed a large population of cells in the ‘monocyte window’ (Figure 3). Based on their similar forward and side scatter properties, the XN-series classified ‘hairy cells’ as monocytes, which resulted in a spurious ‘monocytosis’ flag. No white cell precursor scatterplot was generated by the instrument, suggesting that the white cell precursor channel (which detects abnormal lymphocytes or blasts) was not reflexed. Similarly, in five of six HCL cases evaluated by Tofan et al.^3^, a pseudomonocytosis was generated when automated differential counts were performed on the Sysmex XN-series. This impaired separation of mononuclear cells was ascribed to the larger size and more abundant cytoplasm of the leukaemic cells.^3^

The WBC flagging performance of the XN-series has been evaluated in several studies, including two local studies performed in NHLS laboratories.^8,15,16^ Ramiah et al.^8^ demonstrated sensitivities and specificities of 90% and 96.2% for the ‘abnormal lymphocyte’ flag, and 84.3% and 97% for the ‘monocytosis’ flag. Three samples (1.2%) were erroneously flagged as ‘monocytosis’ where blasts were demonstrated on microscopy.^8^ Schapkaitz et al.^16^ evaluated the performance of the Sysmex XN-9000 and found the white cell differential and white cell precursor channels to be 100% sensitive for detecting abnormal lymphocytes. However, neither of these studies included HCL cases.

The limitations of Sysmex automated FBC haematology analysers in flagging abnormal results in HCL have not been widely reported. Furundarena et al.^11^ compared the performance of Sysmex XN and XE-5000 analysers in oncohaematologic patients and concluded that both instruments must improve flagging in HCL. Both analysers only generated one flag (‘Abn Lympho/Blasts?’) in one out of five HCL samples included in their study.^11^

Considering the rarity of HCL, this neoplasm may be underrepresented in the verification studies of XN-series analysers that have been performed to date. Further studies are needed to evaluate the performance of the XN-series, specifically in HCL.

### Conclusion

Monocytopenia may prompt early investigation for HCL in the right clinical scenario. However, haematology analysers, such as the Sysmex XN-series, that rely on cytochemistry with fluorescence flow cytometry may artifactually report ‘monocytosis’ in HCL. With the nationwide introduction of the Sysmex XN-series in NHLS laboratories, this case serves as a reminder that a manual differential count and morphologic review of suspicious results remain important in the era of automation.

## References

[1] Cross M, Dearden C. Hairy cell leukaemia. Curr Oncol Rep. 2020;22(5):42. 10.1007/s11912-020-00911-0 32297104

[2] Parry-Jones N, Joshi A, Forconi F, Dearden C; BSH Guidelines Committee. Guideline for diagnosis and management of hairy cell leukaemia (HCL) and hairy cell variant (HCL-V). Br J Haematol. 2020;191(5):730–737. 10.1111/bjh.17055 33053222

[3] Tofan L, Piqueras M, Fuster O, et al. Monocytopenia in hairy cell leukemia, a difficult feature to detect using sysmex XN series hematology analyzer. Int J Fam Commun Med. 2022;6(3):94–97. 10.15406/ijfcm.2022.06.00271

[4] Bigorra L, Larriba I, Gutiérrez-Gallego R. The hairy cell leukaemia oxymoron: Monocytotic monocytopenia. Clin Chem Lab Med. 2020;59(3):e111–e115. 10.1515/cclm-2020-0253 32383689

[5] Lynch DT, Hall J, Foucar K. How I investigate monocytosis. Int J Lab Hem. 2018;40:107–114. 10.1111/ijlh.12776 29345409

[6] Troussard X, Maître E, Paillassa J. Hairy cell leukemia 2024: Update on diagnosis, risk-stratification, and treatment-Annual updates in hematological malignancies. Am J Hematol. 2024;99(4):679–696. 10.1002/ajh.27240 38440808

[7] National Health Laboratory Service (NHLS). Placement of Full Blood Count Haematology Analysers for NW/FS, Limp/MP, GP, KZN, EC and WC/NC Regions including Service and Maintenance for a Period of Five (5) Years [homepage on the Internet]. Roche Diagnostics (Pty) Ltd; 2022 [cited 2024 Jul 12]. Available from: https://nhlsqa1.nhls.ac.za/placement-of-full-blood-count-haematology-analysers-for-nw-fs-limp-mp-gp-kzn-ec-and-wc-nc-regions-including-service-and-maintenance-for-a-period-of-five-5-years-6/

[8] Ramiah J, Pillay D, Rapiti N. Performance of the automated Sysmex XN-3000 analyser for detecting white blood cell abnormalities in South Africa. Afr J Lab Med. 2023;12(1):2140. 10.4102/ajlm.v12i1.2140 38094982 PMC10716597

[9] Joubert J, Weyers R, Raubenheimer JE. Reducing unnecessary blood smear examinations: Can Sysmex blood cell analysers help? Med Technol SA. 2014;28(1):6–12.

[10] Grever MR, Abdel-Wahab O, Andritsos LA, et al. Consensus guidelines for the diagnosis and management of patients with classic hairy cell leukemia. Blood. 2017;129(5):553–560. 10.1182/blood-2016-01-689422 27903528 PMC5290982

[11] Furundarena JR, Sainz M, Uranga A, et al. Comparison of abnormal cell flagging of the hematology analyzers Sysmex XN and Sysmex XE-5000 in oncohematologic patients. Int J Lab Hematol. 2017;39(1):58–67. 10.1111/ijlh.12575 27981789

[12] Alaggio R, Amador C, Anagnostopoulos I, et al. The 5th edition of the World Health Organization classification of haematolymphoid tumours: Lymphoid neoplasms. Leukemia. 2022;36(7):1720–1748. doi: 10.1038/s41375-022-01620-2. Erratum in: Leukemia. 2023;37(9):1944–1951. https://doi.org/10.1038/s41375-022-01620-2 35732829 PMC9214472

[13] Sysmex Corporation. Chapter 15: p. 466–469. In: Automated Hematology Analyzer XN series Transportation units (XN-9000/XN-9100). Instructions for use. (ver.22). Kobe: Sysmex Corporation; 2021.

[14] Sysmex Corporation. Chapter 5: p. 66–71. In: Automated Hematology Analyzer XN-L series (XN-530/XN-430/XN-330). General Information (ver.5). Kobe: Sysmex Corporation; 2020.

[15] Sysmex South Africa Scientific Customer Services. Customer literature list – White blood cells. 2024(03). 2024 [cited 2024 May 31]. Available from: https://www.sysmex.co.za/academy/library/publications/

[16] Schapkaitz E, Raburabu S. Performance evaluation of the new measurement channels on the automated Sysmex XN-9000 hematology analyzer. Clin Biochem. 2018;53:132–138. 10.1016/j.clinbiochem.2018.01.014 29374555

